# Validation of the Arabic Version of the Maslach Burnout Inventory-HSS Among Tunisian Medical Residents (A-MBI-MR): Factor Structure, Construct Validity, Reliability, and Gender Invariance

**DOI:** 10.3390/healthcare13020173

**Published:** 2025-01-16

**Authors:** Hamdi Henchiri, Amayra Tannoubi, Chayma Harrathi, Ghada Boussayala, Frank Quansah, John Elvis Hagan, Haifa Mechergui, Amr Chaabeni, Taha Chebbi, Tarek Ben Lakhal, Hatem Belhouchet, Ikram Khatrouch, Abdulhafed Mabrouk Gawar, Fairouz Azaiez

**Affiliations:** 1 Higher Institute of Sport, and Physical Education of Sfax, University of Sfax, Sfax 3000, Tunisia; hamdyhenchiri@gmail.com (H.H.); ghadaboussayala@gmail.com (G.B.); fairouz.kyranis@yahoo.com (F.A.); 2 Group for the Study of Development and Social Environment (GEDES), Faculty of Human and Social Science of Sfax, Sfax 3000, Tunisia; 3 Occupational Medicine and Professional Pathologies Department, Gafsa Regional Hospital, Gafsa 2100, Tunisia; harrathi.chayma@gmail.com; 4 Higher Institute of Sport and Physical Education of Gafsa, University of Gafsa, Gafsa 2100, Tunisia; atanoubi@gmail.com (A.T.); tahachebbi1234@gmail.com (T.C.); 5 Faculty of Medicine of Monastir, Monastir 5000, Tunisia; amrch97@gmail.com; 6 Department of Educational Foundations, University of Education, Winneba P.O. Box 25, Ghana; fquansah@uew.edu.gh; 7 Neurocognition and Action-Biomechanics-Research Group, Faculty of Psychology and Sports Science, Bielefeld University, Postfach 10 01 31, 33501 Bielefeld, Germany; 8 Department of Health, Physical Education and Recreation, University of Cape Coast, Cape Coast PMB TF0494, Ghana; 9 Faculty of Medicine of Tunis, Tunis 1007, Tunisia; haifaamechergui@gmail.com; 10 Faculty of Sciences of Sfax (FSS), University of Sfax, Sfax 3000, Tunisia; tarek.ben-lakhal@uha.fr; 11 Faculty of Sciences and Techniques (FST), University of Haute-Alsace, 68200 Mulhouse, France; 12 Laboratory of Research in Society & Humanities, University Polytechnique Hauts-de-France, F-59313 Valenciennes, France; hatem.belhouchet@uphf.fr; 13 INSA Hauts-de-France, LARSH, F-59313 Valenciennes, France; 14 Laboratory QUARTZ, IUT of Montreuil, Paris 8 University, 93100 Montreuil, France; ikram.khatrouch@yahoo.fr; 15 Faculty of Physical Education and Sport Sciences of Tripoli, Université of Tripoli, Tripoli 13932, Libya; gaw.2022@gmail.com

**Keywords:** Arabic context, burnout, confirmatory factor analysis, gender invariance, medical students, residents

## Abstract

**Background**: Burnout is a major problem for physical and mental health of medical residents. The key for maintaining well-being and quality of care of residents is the assessing tool. The study evaluated the psychometric properties of the Arabic version of the Maslach Burnout Inventory Human Services Survey (MBI-HSS) among Tunisian medical residents by assessing its factor structure, construct validity, reliability, and gender invariance. **Methods**: A total of 552 residents, aged 27.01 ± 1.92, 219 males (39.7%) and 333 females (60.3%) completed the Arabic version of the A-MBI-MR. The exploratory (EFA) and confirmatory factor (CFA) analyses were performed to identify the factor structure, with assessments of internal consistency of the model, including gender measurement invariance. **Results**: The results indicate a high average variance extracted (AVE > 0.50) and factor loading of the scale, signifying robust construct validity and clearly suggesting that the items serve as essential indicators for assessing several dimensions of burnout. The reliability analysis demonstrates excellent and acceptable internal consistency across all areas of emotional exhaustion, personal accomplishment, and depersonalization (Cronbach’s α = 0.95, 0.98, and 0.871) respectively. The CFA confirmed the three-factor structure of the A-MBI-MR, with fit indices indicating an adequate model fit: CFI = 0.945, TLI = 0.938, GFI = 0.951, RMSEA = 0.074, RMSEA CI (0.066, 0.081), RMSEA *p*-value= 0.000, SRMR = 0.044. Results from the measurement invariance analysis of the MBI scale demonstrated robust invariance between male and female participants. **Conclusions**: The MBI-MR in Arabic for medical residents is reliable, valid, and effective for measuring burnout levels in Arabic-speaking regions.

## 1. Introduction

Doctors usually undergo a mandatory transitional year at the beginning of their career known as “residency”. This transition is the bridge that connects the medical student still enthusiastic, energetic, and fresh to the professional world, with all the responsibilities and commitments it entails.

Resident doctors are expected to rotate through various surgical and medical departments, gaining experience with the most common and serious emergencies. Notably, these responsibilities and pressures in work can have a negative impact on the mental health, well-being, and quality of life of medical residents [1,2,3]. According to research, residents are especially vulnerable to the effects of chronic stress, anxiety, and burnout [4,5,6,7,8,9].

Residents, at the start of their careers, often face a variety of challenges that cause them to experience stress levels that are far above the norm. They must juggle extremely long working hours, heavy workloads, and high expectations from their superiors and hospital [10,11]. They experience a state of intense mental and emotional fatigue that, over time, impairs their ability to perform professional tasks [12,13]. Residents also experience increased anxiety because they are constantly required to make critical decisions under pressure, often without the time or support needed to fully reflect on each situation [14,15,16]. The constant feeling of being evaluated, not measuring up, or making mistakes that could have serious consequences for patients fosters a pervasive sense of insecurity. The prolonged stress associated with these working conditions can cause burnout [4,5,17,18]. Because it is a persistent stressor that drains psychological and emotional resources, insecurity—whether emotional or job-related, is closely associated with burnout [19]. Burnout’s main components—emotional exhaustion, depersonalization, and decreased personal accomplishment—are exacerbated by job uncertainty, such as fear of losing one’s position or career instability [20]. Similar to self-doubt, emotional insecurity makes stress worse and impairs coping mechanisms [19]. This relationship is supported by research, which demonstrates that insecurity makes people more susceptible to burnout by increasing stress which impedes the recovery of resources [19,21,22].

In the 11th revision of the *International Classification of Disease* published by the World Health Organization, burnout was recognized as an occupational phenomenon [23]. Depersonalization, emotional exhaustion, and low personal accomplishment are the characteristics of this phenomenon [20]. Emotional exhaustion (EE) is defined as a state of being emotionally drained and depleted of emotional resources due to excessive demands and prolonged stress at work [24]. Depersonalization (DP) is a detachment from or negative, callous attitude toward colleagues or the work itself [20]. Personal accomplishment (PA) is a diminished sense of professional effectiveness and achievement, characterized by a feeling of incompetence and lack of productivity [25]. Meanwhile, burnout can develop in the work environment, and for this, the residents are showing different psychological and physical disorders that can also influence their social life. The nature of their work makes these issues likely to cause changes in their personality and affect their capacity to focus [26,27,28], particularly in cases of managing nervous patients and dealing with administrative personnel. Ignoring tiredness for too long without treatment might cause major medical issues including sleep disorders and heart diseases, also in many times, studies have shown that residents culminate in tentative of suicide [29,30,31,32,33].

In Tunisia, studies have already proven that burnout seems to be a problem for residents. Zid et al. [34] found that 17.14% of residents had severe burnout. Also, Haouari et al. [35] indicate that 12% of residents had scores indicating severe personal burnout, while 20% had scores indicating moderate personal burnout. Moreover, a study conducted by Zouari et al. [36] among 50 Tunisian medical residents revealed that 94.6% of the participants had a score in favor of burnout, of which 19.6% was severe, while in another study among psychiatry residents, 37.5% of the participants met the criteria for severe burnout. In addition, Feki et al. [37] also discovered high emotional fatigue is experienced by over half (58.2%) of Tunisian medical residents, while high depersonalization was found in 62.5% and low personal accomplishment in 12.5% of cases.

To maintain residents’ health and the quality of services provided to patients, effective measurement tools must be used on a regular basis to assess the levels of burnout that the residents are experiencing. These tools assist employees in assessing the impact of their work environment on their mental health. One of the most widely used tools for measuring and evaluating burnout is the Maslach Burnout Inventory-Health Care Professionals (MBI-HSS) [38].

The 22-question Maslach Burnout Inventory contains three dimensions, which assess emotional exhaustion (EE), depersonalization (DP), and a sense of personal accomplishment (PA), and the MBI service survey is used in various cultural contexts and language [39,40,41,42,43]; however, to the best of our current knowledge, there is a gap in the literature regarding the validation of the MBI in Arabic-speaking countries which is the case in Tunisia. The Maslach Burnout Inventory (MBI) was selected as the main instrument for this study despite the existence of other burnout measurement tools (e.g., the Copenhagen Burnout Inventory (CBI) [44], and the Oldenburg Burnout Inventory (OBI) [45]) because of its extensive record of validation, reliability, and widespread use in burnout research, especially in healthcare settings. Since its inception, the MBI has established itself as the gold standard for measuring burnout, offering a solid framework for evaluating the three main dimensions of burnout [38,46]. Researchers are concerned about the availability of well-calibrated tools for examining burnout experiences across time, given the important role that mental health plays in the workplace. For the consistent assessment of items and elements that are not performing effectively, constant calibration and modification are essential [47]. It is crucial to regularly assess the components and variables that are unsuccessful. It is also essential to understand the MBI model’s framework in the context of the Arab and Tunisian regions. People with poor English language skills, for example, can misunderstand the questions and provide imprecise answers [48]. The MBI-MR’s transcultural adaptation makes it easier to understand the inventory’s accuracy in a variety of cultural situations. Further, because language and culture are closely related, different people will interpret the MBI items differently depending on the language used and the social context. People generally have a strong emotional attachment to their mother tongue, which makes them more likely to be enthusiastic when answering a survey in their mother tongue [49]. Thus, the study’s objective was to validate the Maslach Burnout Inventory (MBI) among medical residents in Tunisia by assessing its psychometric properties, focusing on its validity, reliability, factor structure, and gender invariance.

## 2. Materials and Methods

### 2.1. Participants

A total of 552 medical students participated in this study. The data present demographic insights on marital status, and study choice among male and female participants. The age ranged from 24 to 31 years old (M = 27.1 ± 1.92 years). A significant proportion are single, with 31% of males and 46.2% of females, while married individuals account for 8.7% of males and 14.1% of females, indicating a predominantly unmarried population.

The study included medical residents with at least five months of clinical experience. Individuals who did not enroll in a residency program, with insufficient experience, or were unable to finish the data collection were excluded from the study.

### 2.2. Measures

Inventory of the Maslach Burnout (MBI-HSS). The MBI-HSS is a self-administered measure of attitudes and feelings toward work that consists of affirmative sentences [20]. Emotional exhaustion, depersonalization, and lack of personal accomplishment are their three dimensions. The Maslach Inventory is the one that is most frequently cited in the literature. There are 22 items in this survey, which are divided into three dimensions: depersonalization (5 items), emotional exhaustion (9 items), and (reduced) personal accomplishment (8 items). A 7-point Likert scale was used, with 0 (never) and 6 (always).

### 2.3. Procedure and Ethical Statement

The study was carried out in four Tunisian university hospitals: Tunis, Sousse, Monastir, and Sfax. The questionnaire included basic socio-demographic variables, and the participation of 552 residents was voluntary. Data were collected during the academic year 2024.

The study was conducted after approval from the ethics committees of the local committee of the Higher Institute of Sport and Physical Education of Kef, Tunisia, with reference number Sp-0030⁄2024 dated 10 February 2024. The study was carried out in accordance with the latest Declaration of Helsinki [50]. Students were informed that filling in the questionnaires was voluntary, that their answers would be treated confidentially, and that there would be no negative consequences for not participating in the study. All participants gave written informed consent. They were also informed that they could withdraw their consent at any time, without having to give a reason.

### 2.4. Translation and Cross-Cultural Adaptation of the Instrument

The Maslach Burnout Inventory-Human Services Survey (MBI-HSS) was prepared in the Arabic language through systematic and valid procedures. Cultural sensitivity and cross-cultural adaptation was applied to the English version of the instrument to improve its use in the Arabic-speaking population following the international test commission’s guidelines for cross-cultural test adaptation [51]. As already mentioned, the first stage began when the two bilingual translators with psychological assessment experience provided forward translations of the items. After this stage, there was a reconciliation stage to sort out the differences that emerged during the translation stage. An independent translator broached back-translation of the work to highlight probable deficiencies within the translation. The version obtained was presented to a qualified psychology and culture panel for appropriateness and comprehensiveness. Last but not the least, the prepared scale was pretested among Arabic-speaking physicians in their residency training and refined according to the participants’ responses. Such a systematic process helps guarantee that the Arabic version of MBI-MR is not only a direct translation but also an adaptation into the social-cultural context and perspective.

### 2.5. Statistical Analysis

The psychometric properties of the Arabic-Maslach Burnout Inventory for Medical Resident (A-MBI-MR) were assessed using numerous analyses utilizing the statistical software Jamovi 2.3. for the descriptive and exploratory factor analysis (EFA) and AMOS to assess the confirmatory factor analysis (CFA). After confirming the normality of the data distribution by assessing the skewness and kurtosis values, the internal consistency of the scale was assessed by calculating the Cronbach’s α and the McDonald’s ω coefficient [52]. An EFA and CFA were performed to check the validity of the instrument. The adequacy of the sample was evaluated using the Kaiser–Meyer–Olkin (KMO) measure and Bartlett’s test of sphericity. The criterion employed was a number of components exhibiting eigenvalues exceeding 1 [53]. 

The factor structure of the measure A-MBI-MR was initially assessed by EFA. A CFA was subsequently conducted to assess factor loadings, evaluating the strength of relationships between observed variables and latent factors, with loadings of 0.60 or higher deemed acceptable indicators of robust construct validity [54]. Model fit indices, such as the Comparative Fit Index (CFI) and Tucker–Lewis Index (TLI), Goodness of Fit index (GFI), and the Root Mean Square Error of Approximation (RMSEA) were assessed. Hu and Bentler suggested RMSEA values below 0.08 for adequate fit and values above 0.95 for the TLI and CFI [54]. A gender invariance measurement was conducted to ensure that the tool is equally valid and interpretable for males and females [55].

## 3. Results

### 3.1. Descriptive Statistics and Normality

The mean, standard deviation, skewness, and kurtosis estimates of the items have been provided in Table 1.

As shown in Table 1, the distribution appeared to be normal given the measures of skewness [−1, 1] and kurtosis [−2, 2].

### 3.2. Factor Structure of the A-MBI-MR

**Figure 1:**
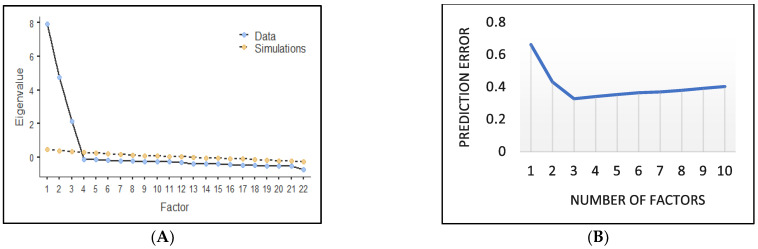
(**A**) Scree plot (with parallel analysis). (**B**) Out-of-sample prediction error.

The study first sought to understand the factor structure of the MBI. Using three analytical approaches (data suitability, number of factors, and factor rotation), the factor structure of the MBI was explored. Figure 1A,B, and Table 2.

#### 3.2.1. Data Suitability

Preliminary analysis prior to conducting the EFA showed that the data generally met the eligibility criteria for the analysis. The Kaiser–Meyer–Olkin (KMO) [56] estimates for all the items were greater than 0.60 with a range between 0.82 and 0.942 (see Table 2). The overall measure of sampling adequacy value of 0.911 also confirms the suitability of the data. The Bartlett’s test statistic [56] showed a significant result, Χ^2^ (231) = 7517.732, *p <* 0.001. The sample size of 250 is also considered adequate for performing the EFA [57].

#### 3.2.2. Number of Factors

Closely observing Figure 1A,B provides information about the number of factors to retain for the A-MBI-MR. First, the number of factors associated with eigenvalues greater than 1 is three (see Figure 1A). Secondly, the parallel analysis [58] outcome from Figure 1A revealed that the point where the simulation data becomes greater than the observed data happens after the third factor. Further analysis using the out-of-sample prediction error reveals that after the prediction errors decrease to the third factor and start increasing again, suggesting that three factors are appropriate (see Figure 1B).

Following the acceptance of stability of three factors across the multiple procedures, the factor rotation provided much information into which specific factor the items strongly load unto with their associated uniqueness (see Table 2). All the indicators were within the acceptable range. The analysis revealed that the three-factor structure and their associated items showing the strongest load are consistent with the overall structure of the original MBI (i.e., Personal accomplishment for F1, emotional exhaustion for F2, and depersonalization for F3).

**Table 2:** Factor rotation.

	F1	F2	F3	Uniqueness	MSA
ITEM9	0.998			0.044	0.942
ITEM12	0.985			0.049	0.828
ITEM7	0.983			0.045	0.905
ITEM4	0.980			0.066	0.894
ITEM19	0.980			0.050	0.903
ITEM17	0.956			0.066	0.876
ITEM21	0.945			0.071	0.918
ITEM18	0.858			0.243	0.940
ITEM1		0.802		0.300	0.914
ITEM6		0.802		0.423	0.938
ITEM2		0.782		0.481	0.926
ITEM3		0.777		0.408	0.918
ITEM14		0.767		0.416	0.941
ITEM20		0.711		0.436	0.918
ITEM8		0.707		0.415	0.944
ITEM16		0.695		0.410	0.951
ITEM13		0.687		0.499	0.939
ITEM5			0.981	0.009	0.828
ITEM10			0.929	0.164	0.843
ITEM15			0.929	0.115	0.918
ITEM22			0.885	0.237	0.866
ITEM11			0.816	0.358	0.859
Overall MSA					0.911

F1: Factor 1; F2: Factor 2; F3: Factor 3; MSA—measure of sampling adequacy.

### 3.3. Construct Validity

Following the identification of a three-factor model in the previous analysis, the three-factor first-order CFA was fitted. To address the construct validity of the A-MBI-MR, indicators such as factor loading, AVE, and factor covariances were studied.

#### 3.3.1. Model Fit

Almost all the model fit indices showed an overall good fit. The CFI, GFI, and TLI estimates showed values greater than 0.90 indicating a good fit [54] (CFI = 0.945, TLI = 0.938, GFI = 0.951). The SRMR was less than 0.08 and also indicated a good fit (SRMR = 0.044) whereas the RMSEA values falling between 0.05 and 0.08 showed acceptable fit indices (RMSEA = 0.074).

#### 3.3.2. Average Variance Extracted

The analysis on the AVE [59] revealed that about 85.9 percent of the variances in the items (measuring personal accomplishment) is explained by the underlying sub-dimension. The results also showed that 66.8 percent and 58.9 percent of the variations in the emotional exhaustion and depersonalization domains’ items were explained by the domains, respectively. The AVE values for all the domains were greater than 0.50, indicating that all the items represented their respective construct well, thereby reflecting strong construct validity.

#### 3.3.3. Factor Loadings

**Figure 2:**
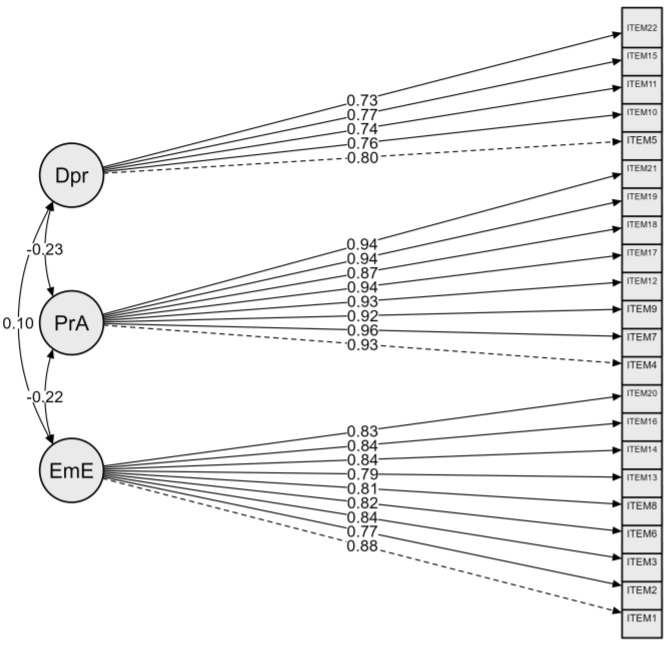
The final CFA model for the three-factor A-MBI-MR. Model indicators: Χ^2^ (206) = 545.770, *p* < 0.001, log-likelihood = −9626.129, CFI = 0.945, TLI = 0.938, GFI = 0.951, RMSEA = 0.074 RMSEA CI (0.066, 0.081), RMSEA *p*-value = 0.000, SRMR = 0.044. Average variance extracted: emotional exhaustion (EmE) = 0.668; personal accomplishment (PrA) = 0.859; depersonalization (Dpr) = 0.589.

The factor loadings were generally acceptable since they were all greater than 0.50 [60]. The emotional exhaustion domain showed strong factors loading with values ranging between 0.74 and 0.87. For depersonalization, the lowest factor loading was 0.73 with the highest factor loading being 0.83. The items for personal accomplishment demonstrated strong factor loadings on their respective factors ranging between 0.86 and 0.95. The high factor loading loadings recorded show evidence of construct validity present (see Figure 2).

#### 3.3.4. Factor Covariance

The factor covariance analysis showed that the personal accomplishment factor is negatively associated with emotional exhaustion (*r =* 0.22) and depersonalization (*r =* 0.23). A positive relationship was found between emotional exhaustion and depersonalization (*r =* 0.10).

### 3.4. Reliability Analysis

Further analysis was carried out to assess the reliability of the MBI using both McDonald’s Omega and Cronbach’s alpha estimation procedures [61]. The reliability analysis results are presented in Table 3.

As presented in Table 3, the reliability indices for the emotional exhaustion domain revealed exceptional internal consistency suggesting that the items on the domain are stable in terms of measuring the same trait (ω = 0.950, α = 0.949). Similarly, the reliability estimates for the personal accomplishment domain showed excellent internal consistency indicating high precision of the items in measuring the construct (ω = 0.980, α = 0.980). Although the depersonalization dimension also showed high internal consistency, their reliability indices were slightly lower than the other two domains (ω = 0.871, α = 0.853).

### 3.5. Gender Measurement Invariance

The research also sought to assess the gender measurement invariance [55] of the A-MBI-MR. The detailed analysis outcome is displayed in Table 4.

The measurement invariance results, as presented in Table 4, suggested that the MBI demonstrated strong invariance across both genders. Particularly, the configural invariance estimates indicated the three-factor structure of the MBI is consistent for both male and female respondents on the MBI (CFI = 0.947, TLI = 0.941, RMSEA = 0.077, SRMR = 0.038). The metric invariance showed excellent fit indicators for the CFI, TLI, and RMSEA, indicating a slightly better improvement over the configural invariance model. The results from the metric invariance confirm equal factor loadings across both genders (CFI = 0.948, TLI = 0.944, RMSEA = 0.075, SRMR = 0.040). The scalar invariance showed a good fit indicating that the intercepts are equal across both genders, recording slight changes in the indicators (CFI = 0.948, TLI = 0.947, RMSEA = 0.073, SRMR = 0.040).

## 4. Discussion

The study validated the Maslach Burnout Inventory-HSS among Tunisian residents (A-MBI-MR, see Appendix A) by assessing its factor structure, construct validity, reliability, and gender invariance.

The findings demonstrate high AVE and factor loading of the scale reflect strong construct validity of the MBI and a clear indication that the items act as core proxies in the measurement of separate aspects of burnout. Personal accomplishment had high factor loadings and AVE estimates, followed by emotional exhaustion and depersonalization factors. This finding reflects the key role played by personal accomplishment in the measurement of burnout among the residents sampled in this research. It is therefore anticipated that the personal accomplishment factor had a negative association with emotional exhaustion and depersonalization. These results are in line with previous studies that have evaluated the factor structure of the original scale in several professional fields [62,63,64].

Further, the reliability analysis shows high and acceptable internal consistency across all the domains of emotional exhaustion, personal accomplishment, and depersonalization which indicates reliable scores from the MBI utilization in burnout diagnosis and intervention assessment. The findings suggest that the items dependably measure their underlying burnout sub-dimension, thereby improving the confidence in the utilization of the MBI. These findings confirm those found in previous studies that validated the scale in different languages [39,40,65,66]. The high reliability evidence found in each of the sub-scales supports the appropriateness of the items by accurately scaling individuals into the levels of emotional exhaustion, personal accomplishment, and depersonalization, distinctively. This evidence reinforces the need for practitioners who utilize the MBI to provide targeted interventions for individuals based on which dimension they receive high scores on.

The findings from the measurement invariance analysis of the MBI scale showed strong invariance across both male and female students. These findings have considerable implications. First, configural invariance established indicates that both male and female students had a similar conceptualization/manifestation of burnout, specifically, as personal accomplishment, emotional exhaustion, and depersonalization. Hoff and lee [67] conducted a systemic review of burnout and physician gender using 45 studies from 2010 to 2019 and found that burnout was significant for both male and female physicians. Additionally, the association between the proxies for measuring the burnout dimensions and their underlying sub-domain appear equivalent across both genders [68]. This understanding supports a comparison of correlation and regression slopes among both genders in any analysis that involves the use of the MBI. Thus, it is legitimate for meaningful comparison of burnout mean scores (on the MBI) for male and female respondents. Hence, any variations observed reveal true differences in real-life or practical terms and not due to errors of measurement. Therefore, the utilization of the MBI has limited measurement bias whether being used by male or female respondents [69]. This notion translates to fairness in the estimation of burnout levels of individuals who are administered the MBI for burnout interventions.

### 4.1. Theoretical Implications

The validation study of the Maslach Burnout Inventory (A-MBI-MR) in Arabic provides several interesting benefits. The results’ statistical power and reliability are improved by the comparatively high sample size. This procedure allows for a more thorough examination of the scale’s construct validity, factor structure, and reliability in this group. The study also offers significant cross-cultural validity of the A-MBI-MR, which helps make it applicable in Arabic-speaking nations. The procedure is also helpful when it comes to addressing burnout among medical residents in the area. By using well-established psychometric techniques, such as measurement invariance testing and factor analysis, the results are more credible and the scale’s consistency across sample groups is guaranteed.

### 4.2. Practical Implications

The study fills gaps in the literature about the well-being of healthcare professionals by offering a critical assessment of burnout levels among medical residents in Tunisia. Lastly, by concentrating on a particular and pertinent population, the study provides useful insights for enhancing resident assistance and mental health treatments in Tunisia’s medical education programs.

## 5. Limitation

The Maslach Burnout Inventory (A-MBI-HSS) Arabic version was validated among Tunisian medical residents. However, there are several limitations. Initially, the cross-sectional form of the study precludes the establishment of a causal relationship between influencing factors and burnout. It is also possible that the findings may not be applicable to medical residents outside of the Arab world or to medical professionals elsewhere. Self-reported data expose researchers to potential biases such as social desirability bias, and cultural variations in burnout perception may restrict the scale’s usage in different Arabic-speaking contexts. The authors were able to mitigate common method bias by protecting participant privacy, keeping predictors and outcomes apart throughout time, and employing a neutral questionnaire design. To further reduce common method bias and improve the dependability of burnout studies, future research should consider multi-source data gathering, longitudinal designs, and sophisticated statistical methodologies.

The results could also be impacted by unaccounted-for confounding factors including work environment and personal traits, suggesting that the sample could not accurately reflect the total population of Tunisian medical interns. While the study looked at the measure’s gender-specific invariance, more research is needed to ensure the scale’s stability across different demographic groups. Some critics contend that the MBI under-represents the contribution of organizational and structural elements to burnout. These drawbacks highlight how crucial it is to incorporate qualitative information or other tools to the MBI to have a more comprehensive knowledge of burnout. Hence, future research could explore the concurrent or discriminant validity of the A-MBI-MR. The duration of data collection and the exclusive focus on medical residents may restrict the variety of healthcare workers’ knowledge regarding burnout.

## 6. Conclusions

The study adapted and validated the MBI-MR in Arabic for medical residents. The results suggest that the scale has a second-order three-factor structure that is suitable for assessing burnout in medical residents. The instrument is reliable and has good construct validity. The invariance analysis revealed no gender differences. The A-MBI-MR is an effective psychometric instrument that can be used to measure burnout levels, including its three dimensions among medical residents in Arabic-speaking regions.

## Figures and Tables

**Table 1:** Mean, standard deviation, skewness, and kurtosis estimates of the items.

	Mean	Std. Deviation	Skewness		Kurtosis	
	Statistic	Statistic	Statistic	Std. Error	Statistic	Std. Error
ITEM 1	3.23	1.781	0.188	0.104	−1.185	0.208
ITEM 2	3.45	1.824	−0.178	0.104	−1.009	0.208
ITEM 3	3.39	1.788	0.035	0.104	−1.134	0.208
ITEM 6	3.51	1.743	−0.129	0.104	−0.988	0.208
ITEM 8	3.43	1.862	−0.209	0.104	−0.978	0.208
ITEM 13	3.37	1.815	−0.093	0.104	−1.053	0.208
ITEM 14	3.45	1.736	−0.082	0.104	−0.937	0.208
ITEM 16	3.35	1.755	0.000	0.104	−1.069	0.208
ITEM 20	3.38	1.743	0.051	0.104	−1.102	0.208
ITEM 4	3.05	1.947	−0.078	0.104	−1.331	0.208
ITEM 7	3.14	1.947	−0.162	0.104	−1.271	0.208
ITEM 9	3.13	1.904	−0.136	0.104	−1.201	0.208
ITEM 12	2.99	1.966	0.001	0.104	−1.348	0.208
ITEM 17	3.20	1.973	−0.221	0.104	−1.275	0.208
ITEM 18	3.27	1.958	−0.265	0.104	−1.238	0.208
ITEM 19	3.24	1.957	−0.254	0.104	−1.189	0.208
ITEM 21	3.14	2.001	−0.059	0.104	−1.323	0.208
ITEM 5	2.86	1.350	−0.096	0.104	−0.672	0.208
ITEM 10	2.95	1.360	−0.286	0.104	−0.517	0.208
ITEM 11	3.00	1.316	−0.221	0.104	−0.582	0.208
ITEM 15	2.89	1.363	−0.174	0.104	−0.648	0.208
ITEM 22	3.03	1.288	−0.136	0.104	−0.621	0.208

**Table 3:** Reliability estimates based on the dimensions of A-MBI-MR.

Dimensions	Estimate	McDonald’s ω	Cronbach’s α
Emotional Exhaustion	Point estimate	0.950	0.949
	95% CI lower bound	0.943	0.943
	95% CI upper bound	0.956	0.956
Personal Accomplishment	Point estimate	0.980	0.980
	95% CI lower bound	0.978	0.978
	95% CI upper bound	0.983	0.983
Depersonalization	Point estimate	0.871	0.871
	95% CI lower bound	0.854	0.853
	95% CI upper bound	0.888	0.887

**Table 4:** Gender measurement invariance results of the A-MBI-MR.

Indicators	Configural Invariance	Metric Invariance	Scalar Invariance
Model Test User Model:			
Test statistic	1084.773	1094.816	1108.364
Degrees of freedom	412	431	450
*p*-value (Chi-square)	0.000	0.000	0.000
Model Test Baseline Model:			
Test statistic	13,195.872	13,195.872	13,195.872
Degrees of freedom	462	462	462
*p*-value (Chi-square)	0.000	0.000	0.000
User Model versus Baseline Model:			
CFI	0.947	0.948	0.948
TLI	0.941	0.944	0.947
RMSEA	0.077	0.075	0.073
RMSEA CI (LL, UL)	0.071, 0.083	0.069, 0.080	0.067, 0.078
*p*-value H_0: RMSEA ≤ 0.050	0.000	0.000	0.000
*p*-value H_0: RMSEA ≥ 0.080	0.185	0.057	0.054
SRMR	0.038	0.040	0.040
Log-likelihood user model (H0)	−17,784.194	−17,789.215	−17,795.990
Log-likelihood unrestricted model (H1)	−17,241.807	−17,241.807	−17,241.807

## Data Availability

The original contributions presented in the study are included in the article. Further inquiries can be directed to the corresponding author.

## Appendix A. Arabic Version of the MBI-MR

الإرهاق العاطفي (EE)	
أشعر بالإرهاق العاطفي بسبب عملي كطبيب مقيم	1
أشعر بالإجهاد في نهاية يوم العمل في المستشفى	2
أشعر بالتعب بمجرد أن أستيقظ في الصباح وأفكر في يوم عمل جديد أو مناوبة جديدة	3
العمل مع المرضى والفِرق الطبية طوال اليوم مرهق بالنسبة لي	6
أشعر بالإرهاق والإحباط بسبب عملي الطبي	8
أشعر بالإحباط بسبب عبء العمل أو تعقيد مسؤولياتي الطبية	13
أشعر أنني أعمل بجد أكبر من اللازم وأضحي بصحتي من أجل العمل	14
التواصل المباشر مع المرضى والفريق الطبي في العمل مرهق للغاية بالنسبة لي	16
أشعر أحيانًا أنني وصلت إلى أقصى طاقتي العاطفية في العمل الطبي	20
Emotional Exhaustion (EE)	
1. I feel emotionally drained by my work as a resident	
2. I feel exhausted at the end of the workday at the hospital	
3. I feel tired as soon as I wake up in the morning and think about a new workday or a new shift	
6. Working with patients and medical teams all day long is exhausting for me	
8. I feel exhausted and frustrated by my medical work	
13. I get frustrated by the workload or the complexity of my medical responsibilities	
14. I feel like I’m working too hard and sacrificing my health for work	
16. Communicating directly with patients and the medical team at work is very stressful for me	
20. I sometimes feel like I’ve reached my emotional limit in medical work	
الإنجاز الشخصي (PA)	
أستطيع أن أفهم بسهولة تصرفات زملائي الأطباء أو المشرفين علي	4
أتمكن من التعامل بنجاح مع مشاكل الآخرين (المرضى، الزملاء، المشرفين)	7
أشعر أنني أؤثر بشكل إيجابي على الآخرين من خلال عملي، سواء على المرضى أو الزملاء	9
أشعر بالحيوية والطاقة تجاه عملي في مجال الطب	12
أجد أنه من السهل خلق جو مريح ومتعاون في بيئة عملي الطبية	17
أشعر بالتحفيز عندما أعمل عن كثب مع زملائي الأطباء المقيمين	18
حققت العديد من الأهداف المجزية في عملي كطبيب مقيم	19
في عملي الطبي، أشعر بالاسترخاء نسبيًا عندما أتعامل مع المشاكل العاطفية للمرضى أو عائلاتهم	21
Personal Accomplishment (PA)	
4. I can easily understand the behavior of my colleagues or my supervisors	
7. I can successfully deal with other people’s issues (patients, colleagues, supervisors)	
9. I feel that I am positively impacting others through my work, both patients and colleagues	
12. I feel energized and enthusiastic about my work in medicine	
17. I find it easy to create a relaxed and collaborative atmosphere in my medical work environment	
18. I feel motivated when I work closely with my fellow residents	
19. I have achieved many rewarding goals in my work as a resident	
21. In my medical work, I feel relatively relaxed when I deal with the emotional issues of patients or their families	
التجرد من الشخصية (DP)	
أشعر أنني أتعامل مع بعض المرضى بشكل غير شخصي، كما لو كانوا مجرد أشياء	5
أصبحت أكثر قسوة تجاه مشاعر المرضى والزملاء منذ أن بدأت تدريبي كطبيب مقيم	10
أخشى أن يجعلني عملي أكثر قسوة عاطفيًا	11
لا أهتم حقًا بما يحدث مع بعض زملائي في المستشفى	15
أشعر أن بعض زملائي يلومونني على جزء من مشاكلهم المهنية	22
Depersonalization (DP)	
5. I feel like I treat some patients impersonally, as if they are just objects	
10. I’ve become more heartless toward the feelings of patients and colleagues since I started my residency training	
11. I’m afraid my work will make me more emotionally callous	
15. I don’t really care what happens with some of my colleagues in the hospital	
22. I feel that some of my colleagues blame me for part of their professional issues	
